# Supplementary figures and images for: Exploring the molecular basis of the genetic correlation between body mass index and brain morphological traits
Source: PLoS Genet. 2025 Apr 10;21(4):e1011658. doi: 10.1371/journal.pgen.1011658 (PMC12048161; doi:10.1371/journal.pgen.1011658)

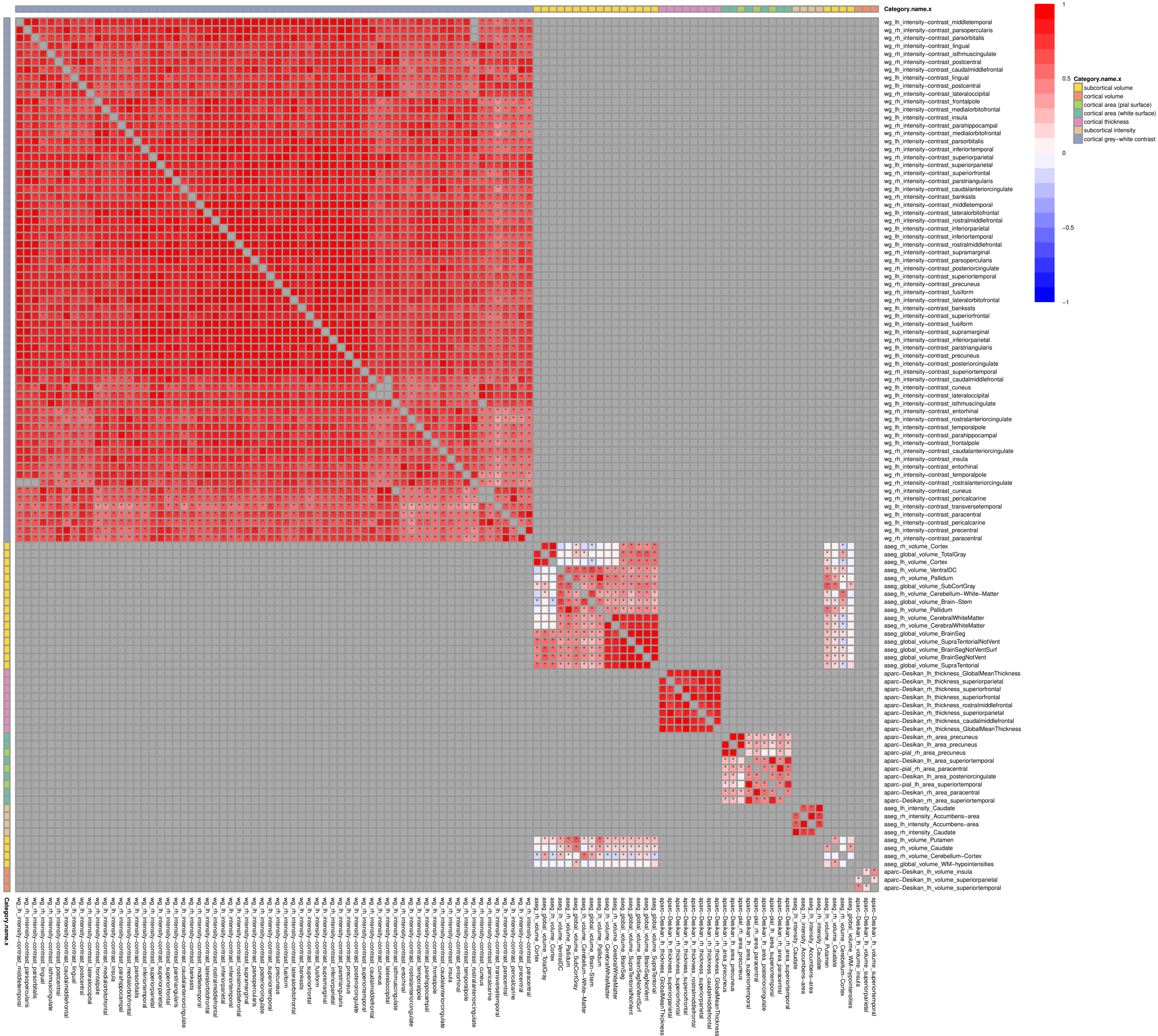

Supplement: S1 Fig — Only the correlations between traits in the same category were computed, plus those between cortical area (pial surface) and cortical area (white surface). ∗, nominally significant (P < 0.05). (PDF) [file pgen.1011658.s004.pdf]

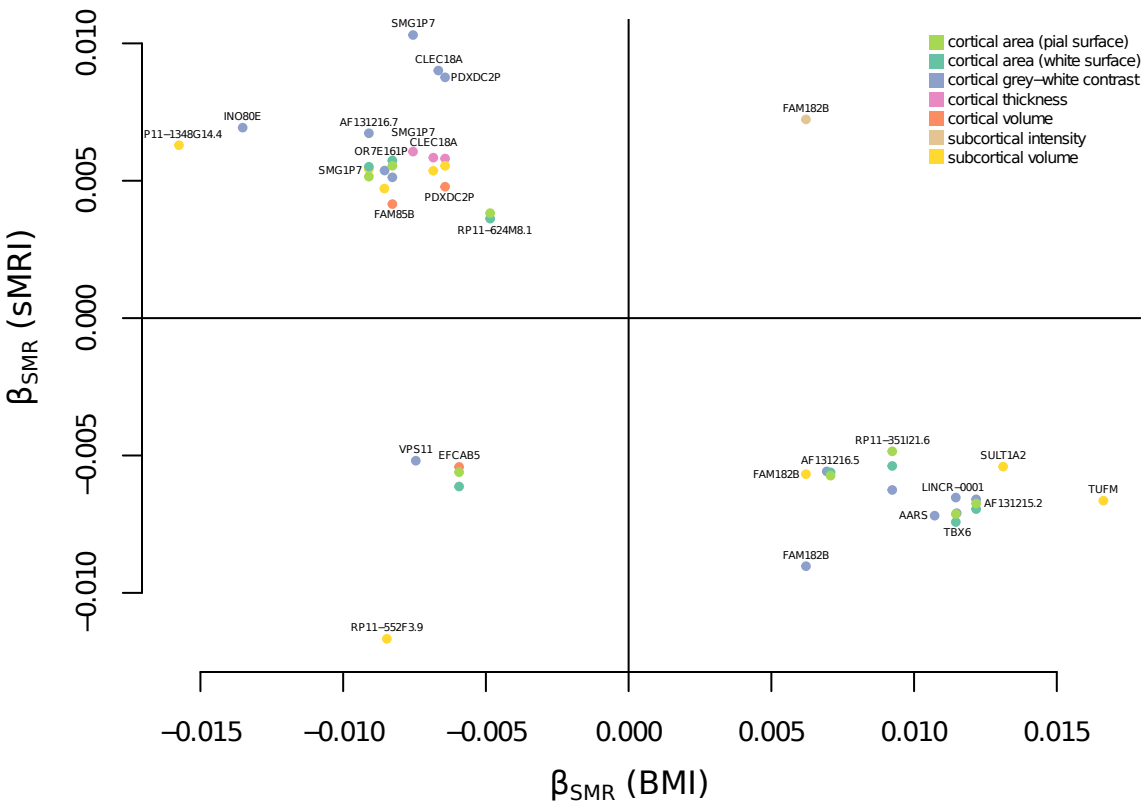

Supplement: S2 Fig — (PDF) [file pgen.1011658.s005.pdf]

**A****RP11-552F3.9**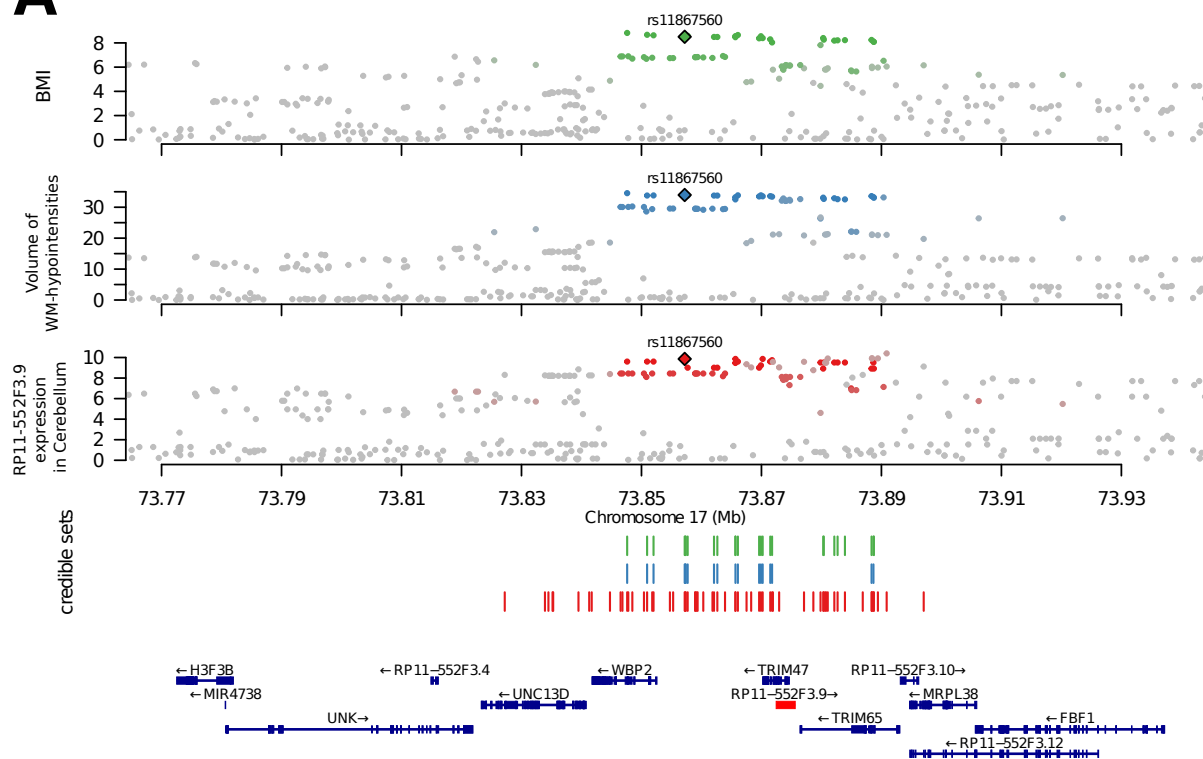**B****RP11-624M8.1**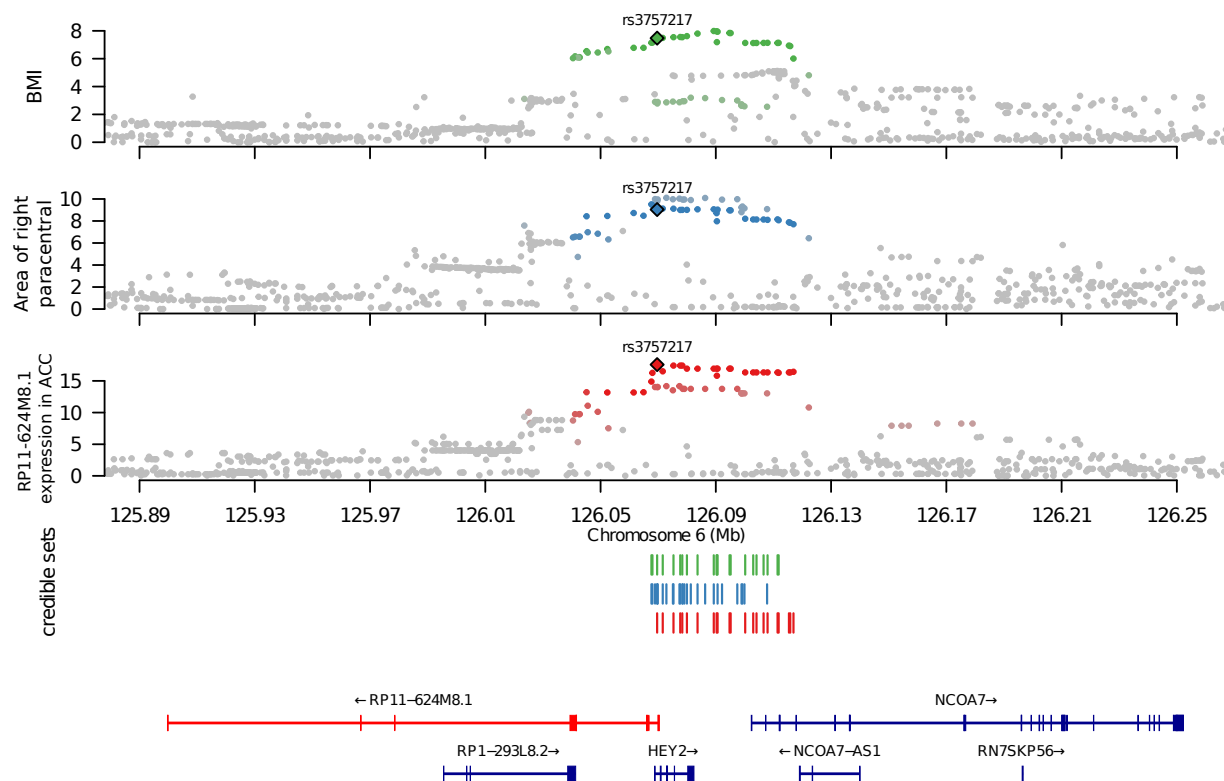

Supplement: S3 Fig — (PDF) [file pgen.1011658.s006.pdf]
