# Supplementary material for: Exploring the molecular basis of the genetic correlation between body mass index and brain morphological traits
Source: PLoS Genet. 2025 Apr 10;21(4):e1011658. doi: 10.1371/journal.pgen.1011658 (PMC12048161; doi:10.1371/journal.pgen.1011658)
Supplement: Attachment — Submitted filename: Rebuttal Daniela PLoS Genet.pdf [file pgen.1011658.s007.pdf]

# Point-by-point response to reviewers

Original reviewer's text is in blue

## Reviewer #1

Fusco et. al, in their manuscript "Exploring the molecular basis of the genetic correlation between body mass index and brain morphological traits" show their exploration of the molecular basis of genetic correlation between BMI and brain morphological traits integrating genomic, transcriptomic, and epigenetic data. The authors first found significant genetic correlations between BMI and brain morphology, primarily negative correlations, then identified 21 genes whose GReX in brain tissue is associated with both BMI and brain traits. TUFM and VPS11 are used as showcase examples. Further the authors did three-way colocalization and fine-mapping and reported four loci. Variant rs7187776 in the TUFM gene's 5′ UTR was highlighted as a potential causal variant influencing BMI and brain structure through changes in chromatin accessibility in microglial cells.

The analysis show novelty and is comprehensive, albeit the following concerns should be addressed:

(1) Why used different INFO score filtration for GWAS summary statistics for BMI and brain traits?

We actually used the same INFO score (0.95) for the filtration of all summary statistics. We better clarified this point in the methods section as follows: "All summary statistics *for IDPs and BMI* were filtered for imputation INFO score ≥ 0.95 and minor allele frequency (MAF) ≥ 0.01."

The other INFO score threshold (0.8) was adopted for individual-level data used for LD matrices, stratified LD scores and GReX, given the greater importance of including a large number of variants in these methods (eg. to assess comprehensive LD scores or to avoid losing expression-predictive variants in GReX models).

(2) Could you elaborate the reasons that you picked TUFM and VPS11 for showcase?

While both HEIDI and coloc-SuSiE are colocalization tests that can distinguish between true pleiotropy and the effects of linkage disequilibrium, it is known [Barbeira et al Nat Commun. 9: 1825 doi:10.1038/s41467-018-03621-1] that they agree only partially. For four genes, out of the 21 selected by HEIDI, three-way colocalization was confirmed by coloc-SuSiE, and these included 2 protein coding genes (*TUFM* and *VPS11*) that we chose to show in detail.

(3) Have you assessed how transferable are these findings to other population? What are the allele frequency of rs7187776 in European and other population?

According to dbSNP (ALFA allele frequency project) the MAF of rs7187776 varies between 0.11 in South Asians and 0.48 in Latin American individuals with mostly European and Native American Ancestry, and is equal to 0.37 in Europeans. Thus the SNP can be considered common in all ascertained populations.

As both genetic and epigenetic fine mapping strongly point to a causal role for this SNP, and the magnitude and direction of causal eQTL effects are known to be consistent across populations [see e.g. Taylor et al. Nature. 2024. doi:10.1038/s41586-024-07708-2] it is reasonable to expect that the findings related to this SNP should be transferable to other populations, although we did not formally check whether this is the case. We have included this information in the Discussion section of the manuscript.

(4) How did you select 435 sMRI traits? Are they purely based on data availability or selected by some criteria?

The dataset from the BIG40 server (https://open.win.ox.ac.uk/ukbiobank/big40/), held by Smith et al. (doi: https://doi.org/10.1038/s41593-021-00826-4), includes 3935 imaging derived phenotypes (IDPs), covering a range of structural, diffusion and functional MRI measurements. Among the more than 1400 structural MRI (sMRI) IDPs, we focused on those generated from Desikan-Killiany cortical parcellation and from subcortical volumetric segmentation (aseg). This selection resulted in a subset of 437 sMRI IDPs. Two specific traits, "aseg_lh_number_HolesBeforeFixing" and "aseg_rh_number_HolesBeforeFixing" were excluded from our analysis as they are technical artifacts produced by segmentation rather than morphological measurements. We have added this information to the Methods section of the manuscript.

(5) Would including pathway analysis for TUPM and VPS11 may be helpful to uncover some biological process that may align with your finding?

Following the reviewer's suggestion, we used the STRING database (https://string-db.org/) to extract the top interactors of these two proteins and their Gene Ontology enrichments. However, these results simply confirmed the known functions of the two proteins (mitochondrial translation for TUFM and vacuolar sorting/endosome/autophagy for VPS11). Therefore we chose not to include these analyses, although we described in some more detail the known functional information about these genes in the Discussion. We have included the results for Biological Process at the end of this document.

(6) Most jargons and abbreviations are well-defined, while some were not at the first occurance. For example, "PPA" didn't defined in Figure 2 legend and main text. In main text of coloc-SuSiE

analysis, posterior probabilities (e.g. PPH3, PPH4), are introduced without introducing the hypothesis.

We fixed these issues in the manuscript.

(7) Make sure the “x/y axis” in figure legend are consistent for italic or not. Typo for “axis” in Figure 4 legend.

We fixed these issues in the manuscript.

(8) Specify version for softwares you used.

We have added this information at the end of the manuscript (“Software used”).

(9) “Method: Individual-level GREx-trait association”: R -> R^2

We actually filtered for the correlation coefficient (not its square) to be greater than 0.1, as recommended e.g. in [Zhou et al Nat Genet.52: 1239 doi:10.1038/s41588-020-0706-2]. This is equivalent to the customary filter R^2 > 0.01, but excluding negative correlations between predicted and actual expression which clearly do not signal good predictive power. We have replaced “R” with “r” in the manuscript and clarified this point.

# Reviewer #2:

The study systematically examines the genetic correlations between body mass index and brain morphological traits using data from the UK Biobank. It identifies negative genetic correlations between BMI and multiple brain structure measurements. Through integrative approaches, including Mendelian randomization and fine-mapping, the research highlights genes, like TUFM, associated with these correlations. A notable finding is that specific genetic variants, such as rs7187776, potentially affect BMI and brain morphology by altering chromatin accessibility in microglial cells. I have such questions:

1.In Figure 1, could you please use subscripts for genetic correlations as is done in the main text?

We fixed the figure as recommended by the reviewer.

2.For Figure 1b, what are the rg values for the points that exceed the left-side x-axis?

We have modified the figure to make all confidence intervals fit entirely in the graph.

3.In Figure 2a, it would be helpful to include a legend to better differentiate it from Figure 2b. Additionally, do you have a threshold?

We have added a legend to Figure 2a, explaining the color coding. The PPA threshold for inclusion in the Chicago plot is 0.5, thus the plot includes only genes passing such threshold.

4.How do you deal with the covariates like age and sex?

As the analysis is based on summary data (for BMI and MRI traits) which were derived by the original authors including sex, age, and genomic PCs as covariates, we did not introduce further corrections. We added to the Methods section details about the covariates used in eQTL analysis and GReX imputation.

# Reviewer #3

The manuscript used several analysis methods to explore the relationships among genetic features, BMI, and sMRI traits. The analyses first demonstrated negative genetic correlations between BMI and most sMRI traits. Then assuming gene expressions are the underlying causes of the genetic correlations. 21 genes were found associated with both traits. Colocalization and epigenetic fine mapping found a variant to be causal.

Results: summary based mendelian randomization
SMR + HEIDI and PrediXcan TWAS both identify genes associated with traits. Will PrediXcan identify different gene sets as the 21 found by SMR?

We have shown that a great majority of the associations found by SMR with BMI and sMRI traits would be nominally significant also using individual-level PrediXcan. Using PrediXcan instead of SMR would thus produce gene sets overlapping, but not identical, to those we found. Our choice of SMR was mainly motivated by the availability of the pairing SMR-HEIDI which allows direct testing of colocalization, which we then refine with coloc.

Are the 21 genes found by SMR causal? Is this the reason why using SMR over PrediXcan?

The gene/trait associations found by both SMR and PrediXcan cannot, in general, be considered causal of the trait as the so-called “exclusion restriction” hypothesis of Mendelian Randomization (i.e. the absence of effects of the variant on the trait that do not go through the expression of the gene) cannot be proven true. When SMR is coupled with HEIDI, it is able to distinguish between true pleiotropy (the same variant influences gene expression and trait) from LD (two different variants in linkage disequilibrium with each other affect gene expression and the trait). Finally, three-way coloc further strengthens the argument for pleiotropy. Thus for our 21 genes, and with greater confidence for the 4 genes found by coloc analysis, we can say that there is a single variant which is causal for gene expression, BMI, and the MRI trait. SMR

*suggests*, but cannot prove, that there is causality between gene expression and the other two traits. We have modified the text in the Abstract, Introduction, and Results sections to convey more precisely these points.

Epigenetic fine mapping

Are there any other learning models can be applied to this context? If so, what is the advantage of gkm-SVM?

Models based on convolutional neural networks, such as Basset [Kelley et al Genome Res. 26: 990. doi:10.1101/gr.200535.115] or Sei [Chen et al. Nat Genet.54: 940 doi:10.1038/s41588-022-01102-2] might in principle be more performant than gkmSVM. However, gkmSVM has the advantage of a much shorter training time, a key issue for us as it needed to be applied to more than 100 cell types identified in the atlas we used [Li et al. Science 382: eadf7044 doi:10.1126/science.adf7044]. We have clarified the motivation behind this choice in the Discussion section of the manuscript.

Colocalization

Explain more on why three way colocalization identified variants are causal?

In each of the cases of three-way colocalization, SuSiE identifies, for each of the three traits, a credible set which contains (with 95% confidence) the causal variant. Then coloc evaluates the posterior probability of the causal variant being the same for each of the three pairwise trait comparisons. Thus when the "same variant" hypothesis (H4 in coloc's terminology) has the highest posterior probability in all the three comparisons, we can confidently state that there is one variant, included in the intersection of the three credible sets, which is causal for the three traits. Note that this analysis does not reveal which variant within the intersection is the causal one, and the individual SNPs named in Fig. 4 and Supplementary Fig. 3 are simply *tagging* the intersection of credible sets. We have added a sentence at the beginning of the subsection "Epigenetic fine-mapping", in the Results, to better clarify the relationship between coloc results and the subsequent epigenetic fine-mapping.

The identification of the individual causal variant within the credible set intersection needs epigenetic fine mapping: In the case of TUFM expression we believe such analysis convincingly identifies rs7187776 as a strong candidate, while the results for the other cases are less clear cut.

# APPENDIX: Gene Ontology (Biological Process) enriched terms among the interactors of TUFM and VPS11

## TUFM

| #term ID | term description | observed gene count | background gene count | strength | signal | FDR |
|---|---|---|---|---|---|---|
| GO:0006412 | Translation | 26 | 389 | 1.7 | 6.66 | 1.80E-40 |
| GO:0032543 | Mitochondrial translation | 20 | 112 | 2.13 | 9.97 | 5.03E-36 |
| GO:0002181 | Cytoplasmic translation | 6 | 123 | 1.57 | 1.71 | 8.68E-06 |
| GO:0042255 | Ribosome assembly | 5 | 60 | 1.8 | 1.83 | 1.20E-05 |
| GO:0042254 | Ribosome biogenesis | 7 | 299 | 1.25 | 1.23 | 5.78E-05 |
| GO:0042273 | Ribosomal large subunit biogenesis | 4 | 74 | 1.61 | 1.06 | 0.0016 |
| GO:0140694 | Non-membrane-bounded organelle assembly | 6 | 314 | 1.16 | 0.87 | 0.0016 |
| GO:0000027 | Ribosomal large subunit assembly | 3 | 25 | 1.96 | 1.02 | 0.0032 |

## VPS11

| #term ID | term description | observed gene count | background gene count | strength | signal | FDR |
|---|---|---|---|---|---|---|
| GO:0006906 | Vesicle fusion | 20 | 102 | 2.17 | 10.29 | 4.57E-36 |
| GO:0016192 | Vesicle-mediated transport | 24 | 1298 | 1.15 | 2.3 | 4.13E-23 |
| GO:0061024 | Membrane organization | 21 | 773 | 1.31 | 2.99 | 4.73E-22 |
| GO:0015031 | Protein transport | 22 | 1180 | 1.15 | 2.23 | 3.57E-20 |
| GO:0046907 | Intracellular transport | 22 | 1365 | 1.09 | 1.97 | 6.59E-19 |
| GO:0006886 | Intracellular protein transport | 18 | 683 | 1.3 | 2.71 | 1.13E-17 |
| GO:0034058 | Endosomal vesicle fusion | 8 | 11 | 2.74 | 6.35 | 2.31E-16 |
| GO:0006810 | Transport | 26 | 3785 | 0.72 | 0.96 | 2.67E-16 |
| GO:0022406 | Membrane docking | 11 | 93 | 1.95 | 4.94 | 3.60E-16 |

| | | | | | | |
|---|---|---|---|---|---|---|
| GO:0035542 | Regulation of SNARE complex assembly | 8 | 14 | 2.64 | 6.07 | 7.25E-16 |
| GO:0007034 | Vacuolar transport | 12 | 164 | 1.74 | 4.18 | 1.08E-15 |
| GO:0048278 | Vesicle docking | 10 | 65 | 2.07 | 5.11 | 1.17E-15 |
| GO:0008333 | Endosome to lysosome transport | 10 | 73 | 2.02 | 4.87 | 3.26E-15 |
| GO:0051641 | Cellular localization | 23 | 2677 | 0.81 | 1.14 | 1.30E-14 |
| GO:0006914 | Autophagy | 12 | 303 | 1.48 | 2.87 | 9.23E-13 |
| GO:0051640 | Organelle localization | 13 | 514 | 1.28 | 2.2 | 1.13E-11 |
| GO:0016197 | Endosomal transport | 10 | 230 | 1.52 | 2.66 | 1.25E-10 |
| GO:0016482 | Cytosolic transport | 9 | 147 | 1.67 | 2.98 | 1.25E-10 |
| GO:0042147 | Retrograde transport, endosome to Golgi | 8 | 87 | 1.84 | 3.3 | 1.40E-10 |
| GO:0007033 | Vacuole organization | 9 | 177 | 1.59 | 2.68 | 5.75E-10 |
| GO:0016236 | Macroautophagy | 9 | 188 | 1.56 | 2.59 | 9.41E-10 |
| GO:0006996 | Organelle organization | 21 | 3470 | 0.66 | 0.78 | 1.66E-09 |
| GO:0048193 | Golgi vesicle transport | 7 | 292 | 1.26 | 1.29 | 3.29E-05 |
| GO:0007040 | Lysosome organization | 5 | 96 | 1.6 | 1.47 | 7.02E-05 |
| GO:0090385 | Phagosome-lysosome fusion | 3 | 14 | 2.21 | 1.37 | 0.00047 |
| GO:0044087 | Regulation of cellular component biogenesis | 9 | 971 | 0.85 | 0.7 | 0.00086 |
| GO:0010256 | Endomembrane system organization | 7 | 552 | 0.98 | 0.75 | 0.0018 |
| GO:0007032 | Endosome organization | 4 | 91 | 1.52 | 0.99 | 0.0021 |
| GO:0006904 | Vesicle docking involved in exocytosis | 3 | 44 | 1.71 | 0.81 | 0.0094 |
| GO:0097352 | Autophagosome maturation | 3 | 47 | 1.68 | 0.78 | 0.0111 |
| GO:0007030 | Golgi organization | 4 | 149 | 1.31 | 0.68 | 0.013 |
| GO:0032880 | Regulation of protein localization | 7 | 852 | 0.79 | 0.45 | 0.0255 |
| GO:0048280 | Vesicle fusion with Golgi apparatus | 2 | 9 | 2.23 | 0.67 | 0.0255 |
| GO:0032456 | Endocytic recycling | 3 | 66 | 1.54 | 0.62 | 0.0272 |

| | | | | | | |
|---|---|---|---|---|---|---|
| GO:0000045 | Autophagosome assembly | 3 | 72 | 1.5 | 0.58 | 0.0337 |
| GO:0090161 | Golgi ribbon formation | 2 | 13 | 2.07 | 0.57 | 0.0439 |
| GO:0032482 | Rab protein signal transduction | 2 | 14 | 2.03 | 0.55 | 0.0489 |
